# Eyelid Phenol Peeling as a Potential Alternative to Surgical Blepharoplasty: A Case Series

**DOI:** 10.1155/crdm/7020601

**Published:** 2025-12-29

**Authors:** Jana Dib El Jalbout, Caroline Silva Pereira, Maiara Onetta da Silva, Mariana Vilhena Ferreira, Nancy Emmanuel, Nelson Maurício Júnior, Roberto Tulli, Ivan Rollemberg

**Affiliations:** ^1^ Department of Dermatology, Lebanese American University Medical Center-Rizk Hospital, Beirut, Lebanon, lau.edu.lb; ^2^ Department of Clinical, Cosmetic and Surgical Dermatology, Human Clinic, Sao Paulo, Brazil; ^3^ Department of Dermatology, Faculty of Medicine of the University of Sao Paulo, Sao Paulo, Brazil, usp.br

**Keywords:** chemical peeling, deep peeling, phenol, skin rejuvenation

## Abstract

Phenol peel is a deep peel used to treat conditions involving the reticular dermis such as scars, deep wrinkles, and lentigos and is an attractive alternative to surgical interventions for the rejuvenation of the eyelids and face. Patients who wish to undergo this procedure should be screened for the presence of any contraindications and should be counseled on the possible complications, which include arrhythmia, skin atrophy, scarring, acne eruption, and infection. We describe the cases of four women who underwent deep phenol peeling for the improvement of wrinkles and aging features around the eyelid area as a conservative alternative to surgical blepharoplasty. Patients were previously healthy with no absolute contraindication for the procedure. They were instructed to use the Kligman formula for 1 month prior to peeling. A nonsteroidal anti‐inflammatory medication was given to the patient 30 mins before starting the procedure. The skin was cleansed with urea foam for 1 min, and excess product was removed with a dry and then a moistened gauze until the skin was clean. A 70% alcohol solution was then used to clean the skin area in which the peeling was desired. Phenol concentrated at 88% was applied using a damp cotton swab over multiple layers, until peeling was achieved. At the end of the procedure, a plastic occlusive mask was used to seal the area, and a postphenol occlusive ointment was applied over the periorbital region. Patients were instructed not to wash the area for 7–10 days and were prescribed Hyabak eye drops for dryness as well as oral analgesics as needed. No complications were reported in all cases. Phenol peeling is an easy, relatively safe and effective deep peeling technique that can be used to achieve the desired aesthetic outcomes in patients who wish to improve aging features and minimize the appearance of deep wrinkles without undergoing surgical intervention.

## 1. Introduction

Chemical peeling, also known as chemexfoliation, is an emerging rejuvenation and skin repairing technique, which involves the application of chemical agents to the skin to peel the damaged epidermis and superficial dermis and allow for re‐epithelization, ultimately giving patients an improved skin appearance. This technique has been gaining more popularity over the past years, owing mostly to its cosmetic versatility, notably in the setting of correcting the effects of aging and sun exposure on the skin such as solar elastosis, seborrheic keratosis, wrinkles, and pores [1], making it an appealing lower‐cost alternative to surgical rejuvenation interventions [2]. Nevertheless, chemical peeling has also been indicated for conditions such as scars, acne, warts, and pigmentary diseases such as melasma, freckles, and postinflammatory hyperpigmentation [3]. Depending on the indication for which it is used, the type of chemical peeling, as well as the chemical agent used, differs. For instance, superficial peeling, which is limited to the epidermis, involves caustic agents such as salicylic acid or tretinoin for conditions such as acne, actinic keratosis, hyperpigmentation, or wrinkling. On the other hand, medium peeling targets the papillary dermis using substances such as trichloroacetic acid (TCA) or glycolic acid, whereas deep peeling involves mainly the use of phenol for the treatment of conditions which involve the reticular dermis such as scars, deep wrinkles, and lentigos, among others [4]. Carbolic acid (C_5_H_5_OH) or phenol is a volatile crystalline aromatic hydrocarbon, used as the mainstay chemical agent of deep chemical peeling in combination with croton oil. The latter is extracted from the plant *Croton tiglium* and has the additive effect of inducing vesicles to the skin even at low doses due to its chemical structure involving a free hydroxyl group [4]. Various formulas containing phenol and croton oil have been developed, with phenol concentrations ranging between 45% and 80%. Histological analysis of skin biopsies subjected to phenol solutions reveals epidermal necrosis surrounded by inflammation. Re‐epithelization of the epidermis followed by dermal deposition of uniform and compact collagen and elastic fibers is typically observed within a week from application [4].

Patients who wish to undergo deep phenol peeling should be screened for absolute contraindications such as cardiac diseases, pregnancy or lactation [4], active infections or current wounds on the site of application, and skin cancers particularly facial melanoma. Moreover, past medical history of inflammatory skin conditions, use of drugs with photosensitizing potential or isotretinoin use in the past year, as well as a history of abnormal wound healing, such as keloid formation, should be looked for [3].

Deep phenol peeling carries recognizable complications with concerns of cardiac, hepatic, renal, and respiratory toxicities in cases of significant systemic absorption and ignorant use [5]. The risk of arrhythmias is particularly considerable in the setting of peeling applied to > 1 cosmetic unit or > 0.5% body surface area [6] or in areas of high‐absorption such as the eyelids [4], warranting therefore careful cardiac monitoring throughout the procedure. Pigmentary changes ranging from acute reactive hyperpigmentation to melanotoxicity resulting in depigmentation of affected or remote skin postpeeling have also been reported [5]. Moreover, cicatricial ectropion or keloids have also been noted as considerable adverse outcomes, particularly when phenol is used on periorbital skin [5]. Acne eruptions have been commonly reported following phenol peel in the setting of preexisting acne as well as in its absence, plausibly due to the excessive sebum production by the newly regenerated skin. Although alarming, incidences of infection postpeeling have been rare due to the antimicrobial effects of phenol particularly against bacteria and fungi [4, 7]. In this report, we present four cases of deep phenol peeling performed as a nonsurgical conservative alternative for traditional surgical blepharoplasty.

## 2. Case Presentation

**Figure 1:**
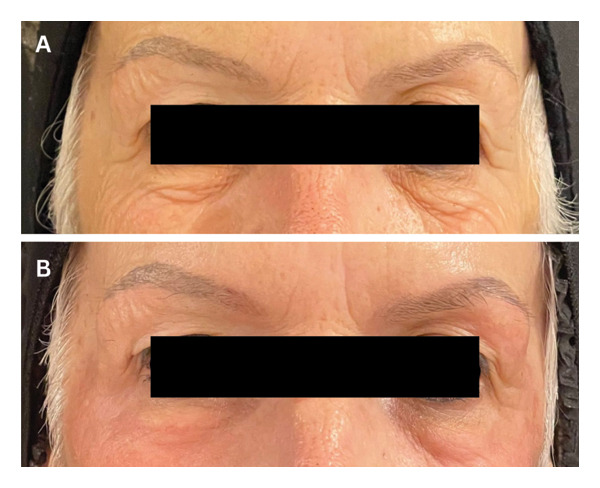
(A) Photograph of a 72‐year‐old lady prepeeling. (B) Photograph of the same patient postpeeling using 88% phenol solution, taken 1 month after the procedure.

**Figure 2:**
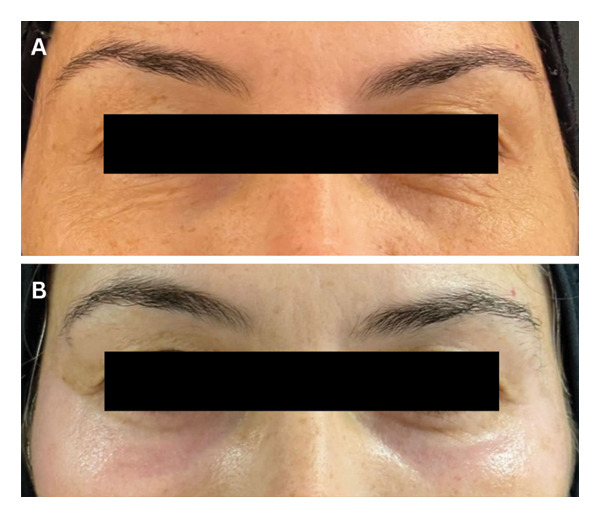
(A) 52‐year‐old lady presenting prepeeling. (B) Photograph of the same patient postpeeling using 88% phenol solution, taken 1 month after the procedure.

We describe the cases of four Brazilian women of European descent, 39, 51, 52 and 72, years old, Fitzpatrick skin types II and III, who presented to the Human Clinic in Sao Paulo, Brazil, for deep phenol peeling for the improvement of periorbital rhytids, ptosis, and other aging features. Their past medical and past surgical history was unremarkable. None of them smoked, and all four wore sunscreen daily. There were no absolute contraindications for the procedure, and patients signed an informed consent after receiving a thorough explanation of the procedure. As preparation prior to the peel, they were instructed to use the Kligman formula (0.1% tretinoin, 0.1% dexamethasone, 5.0% hydroquinone, and hydrophilic ointment) daily for 1 month. A nonsteroid anti‐inflammatory drug, ketorolac trometamol (Toragesic 10 mg, sublingual) was given to the patient 30 mins before starting the procedure, and Regencel ointment (retinol acetate 10,000 IU/g, amino acids 25 g/g, methionine 5 mg/g, and chloramphenicol 5 mg/g) was applied to the eyes. The skin was cleansed with urea foam for 1 minute, and excess was removed with a dry gauze first, followed by a moistened one until the skin was devoid of product. A 70% alcohol solution was used to clean the periorbital skin area in which the peeling was desired. A dry swab was first passed over patients’ skin, and then 88% phenol was applied using a damp cotton swab over multiple layers, with a more humid swab used in every re‐application for a gradual and tolerable build‐up of the product, until peeling was achieved. No complications occurred throughout the four procedures, and patients reported feeling only a burning sensation for around 10 seconds after the first pass of phenol. At the end of the procedure, a plastic occlusive mask was used to seal the area, and a postphenol occlusive ointment was applied over the periorbital region. Patients were instructed not to wash the area for 7–10 days and were prescribed Hyabak eye drops (0.15% sodium hyaluronate) for dryness as well as oral analgesics as needed. Follow‐up was performed 1 week and 1 month after the procedure (Figures 1(a), 1(b), 2(a), and 2(b)), and no complications were reported in all four cases. Evaluator‐based Global Aesthetic Improvement Scale [8] (Table 1) was used to assess postprocedural outcomes, whereby all four patients were given a score of 2 and patients expressed satisfaction with the results achieved.

**Table 1:** Global Aesthetic Improvement Scale (GAIS).

Grade	Description
1	Excellent corrective result
2	Marked improvement
3	Improvement, touch‐up recommended
4	Unchanged compared to the original appearance
5	Worsened compared to the original appearance

## 3. Discussion

Phenol peels have been largely substituted, since their legal introduction into the dermatologic practice in the 1960s [4], by alternative chemical peels such as ≤ 50% TCA with fewer systemic toxicities and decreased risk of cicatricial scarring on periorbital skin due to less aggressive keratocoagulation, and thus higher rates of patient dissatisfaction [9]. Other resurfacing modalities such as voltaic arc dermabrasion [10] and ablative carbon dioxide laser technologies [11] have been applied for the rejuvenation of the skin, treatment of dyschromias, and effacement of aging features with a satisfactory downtime, safety profile and cosmetic result.

Particularly pertinent to the eyelid rejuvenation, phenol peeling was proposed as an adequate adjunct to lower lid blepharoplasty for the treatment of rhytids, pigment dyschromias, and skin laxity, in the absence of considerable fat herniation [12, 13], although blepharopeeling combination may be associated with more complications compared to surgical treatment alone [12]. Moreover, dermal chemical injury produced by phenol peeling translates into significant clinical and histological changes, which can persist for up to 20 years in some patients, resulting in sustained and durable improvements of periorbital skin [5, 14].

In our patients, deep phenol peeling alone was used as an alternative to blepharoplasty for a more conservative rejuvenation of the eyelids. While deep peeling using phenol is generally tolerated and successful in producing desired outcomes, it is not devoid of complications. Various formulations of phenol‐containing solutions are available for use, including Baker–Gordon formula, Venner–Kellson formula, Brown formula, and Litton formula [4]. Prepeeling preparation involves administering antiviral herpes simplex prophylaxis in patients with a history of recurrent herpes starting one day before and 10 days after the procedure. Daily topical 0.1% tretinoin (retin‐A) application to the skin for 3–6 weeks prior to the procedure is recommended to exfoliate the stratum corneum and enable a more equitable penetration and a better absorption of the phenol solution, particularly benefitting patients with oily skin [4, 15]. Moreover, 4%–8% hydroquinone is also recommended in patients with Fitzpatrick skin type III–VI as well as in patients with pigmentary skin diseaseswho are more prone to postpeeling inflammatory hyperpigmentation, since hydroquinone halts melanin synthesis by blocking tyrosinase [15]. As such, in our institution, patients’ skin was prepared using the Kligman formula containing 0.1% tretinoin and 5.0% hydroquinone, 1 month prior to the procedure. Though cardiopulmonary monitoring and intravenous hydration are generally recommended when the surface area being peeled is significant [4], our patients did not undergo any monitoring owing to the small area involved and the decreased risk of systemic absorption linked to toxicity, as in the case series by Gatti et al., where phenol was applied to the eyelids as an adjunct to blepharoplasty without monitoring [13]. It is recommended to instruct patients nevertheless on proper oral hydration with a minimum of 1 L of fluids throughout procedures involving smaller surface areas, not requiring intravenous hydration [6]. Moreover, the peeling of larger surface areas entails the use of regional anesthesia or intravenous sedation for adequate pain control [4]. Nevertheless, sublingual nonsteroidal anti‐inflammatory medication for analgesia, 30 mins prior to the procedure, was sufficient for the purpose of our procedure. It is essential for the skin to be degreased and cleansed from any residual product, with preferred counseling of patients to avoid using any facial creams or make‐up before peeling. The preferred mode of application is using a cotton‐tipped swab to provide precise application; nevertheless, some authors recommend overlying the tip with regular cotton due to the minimal absorption capacity of the cotton swab limiting the amount of product picked up onto the applicator itself [4]. Phenol is then applied on a semidry swab until an “ivory–white” or “gray–white” color of the skin is achieved, over one or multiple layers, depending on the depth of peeling desired. Generally, periorbital or periauricular areas have thinner skin and are considered more anatomically critical, which is why the application of a single layer is advised [16]. In our institution, patients tolerated the application of multiple layers of phenol over the periorbital and under‐eye area using a dampened swab, with more humidity added to the applicator with every re‐application, and a protective eye ointment was applied to prevent any damage or chemical irritation. At the end of the procedure, a plastic occlusive mask with an occlusive ointment was placed over the patient’s face to prevent further unwanted keratocoagulation, and lubricating eye drops were recommended to prevent dryness postprocedure. Patients may experience pain up to 8 hours after peeling until the area achieves complete edema [6], and as such, oral analgesics were prescribed as needed. Certain dermatologists use zinc oxide waterproof tapes, which could be sealed by an orthopedic grip [4]. Typically, for superficial or medium‐depth peeling, gentle cleansing with an antibacterial and antifungal solution such as dilute 1:100 acetic acid is recommended within the first 24 hours after the procedure, with frequent emollient application. After the first day, patients may go back to routine cleansing with nondetergent products, and they are educated about the importance of avoiding sun exposure by using sunscreens, protective clothing, and wide brimmed hats, until at least full‐thickness re‐epithelization is achieved [16]. In our cases, deep peeling was attempted and as such, patients were advised to avoid cleansing the area for at least a week, with similar sun protective measures recommended after the mask is removed.

## Ethics Statement

Ethical approval was not required.

## Consent

Written consent was obtained.

## Disclosure

The authors of this manuscript are grateful for the opportunity to have presented a poster of this work at the EADV Congress 2023.

## Conflicts of Interest

The authors declare no conflicts of interest.

## Author Contributions

Jana Dib El Jalbout: conceptualization, investigation, writing–original draft, review and editing, and visualization.

Nancy Emmanuel: project administration, supervision, and writing–review and editing.

Caroline Silva Pereira: conceptualization, investigation, and methodology.

Maiara Onetta da Silva: conceptualization, investigation, and methodology.

Mariana Vilhena Ferreira: conceptualization, investigation, and methodology.

Nelson Maurício Júnior: conceptualization, investigation, and methodology.

Roberto Tulli: conceptualization, investigation, and methodology.

Ivan Rollemberg: conceptualization, investigation, and methodology.

## Funding

No funding was received for this study.

## Data Availability

The data that support the findings of this study are available from the corresponding author upon reasonable request.

## References

[1] Robles Velasco M. V. , Okubo F. R. , Ribeiro M. E. , Steiner D. , and Bedin V. , Rejuvenescimento da Pele Por Peeling Químico: Enfoque No Peeling de Fenol Facial Skin Rejuvenation by Chemical Peeling: Focus on Phenol Peeling, Anais Brasileiros de Dermatologia. (2004) 79, no. 1, 91–99, 10.1590/s0365-05962004000100011.

[2] Schiedler V. , Chemical Peels for Rejuvenating Eyelids and Face, International Ophthalmology Clinics. (2024) 64, no. 3, 29–40, 10.1097/iio.0000000000000524. 38910503

[3] Poorian B. , Keyhan S. O. , and Chavoshinejad M. , Chemical Peeling, Integrated Procedures in Facial Cosmetic Surgery. (2021) 413–420.

[4] Landau M. , Deep Chemical Peels for Photoaging, Color Atlas of Chemical Peels, 2006, Springer Berlin Heidelberg, Berlin, Heidelberg, 69–89.

[5] De Oliveira Ciaramicolo N. , Bisson G. B. , and Ferreira Júnior O. , Adverse Effects Associated with the Irresponsible Use of Phenol Peeling: Literature Review, Oral Surgery, Oral Medicine, Oral Pathology and Oral Radiology. (2025) 139, no. 2, 161–165, 10.1016/j.oooo.2024.08.018. 39578173

[6] Wambier C. G. , Lee K. C. , Soon S. L. et al., Advanced Chemical Peels: Phenol-Croton Oil Peel, Journal of the American Academy of Dermatology. (August 2019) 81, no. 2, 327–336, 10.1016/j.jaad.2018.11.060, 2-s2.0-85065604510. 30550827

[7] Nikalji N. , Godse K. , Sakhiya J. , Patil S. , and Nadkarni N. , Complications of Medium Depth and Deep Chemical Peels, Journal of Cutaneous and Esthetic Surgery. (2012) 5, no. 4, 254–260, 10.4103/0974-2077.104913. PMC3560165 23378707

[8] Gubanova E. I. and Starovatova P. A. , A Prospective, Comparative, evaluator-Blind Clinical Study Investigating Efficacy and Safety of Two Injection Techniques with Radiesse(®) for the Correction of Skin Changes in Aging Hands, Journal of Cutaneous and Esthetic Surgery. (2015) 8, no. 3, 147–152, 10.4103/0974-2077.167271. PMC4645144 26644738

[9] Dailey R. A. , Gray J. F. , Rubin M. G. et al., Histopathologic Changes of the Eyelid Skin Following Trichloroacetic Acid Chemical Peel, Ophthalmic Plastic and Reconstructive Surgery. (January 1998) 14, no. 1, 9–12, 10.1097/00002341-199801000-00003, 2-s2.0-0031934535. 9513236

[10] Scarano A. , Lorusso F. , Brucoli M. , Lucchina A. G. , Carinci F. , and Mortellaro C. , Upper Eyelid Blepharoplasty With Voltaic Arc Dermabrasion, Journal of Craniofacial Surgery. Ovid Technologies (Wolters Kluwer Health). (2018) 29, no. 8, 2263–2266, 10.1097/scs.0000000000004504, 2-s2.0-85056418561. 29554070

[11] Bonan P. , Fusco I. , Bruscino N. et al., Laser-Assisted Blepharoplasty: An Innovative Safe and Effective Technique, Skin Research and Technology. (2023) 29, no. 5, 10.1111/srt.13351. PMC10189347 37231919

[12] Asilian A. , Shahmoradi Z. , Talakoub M. et al., Evaluation of Combination Therapy With Peeling Added to Minimal Invasive Blepharoplasty in Lower Eyelid Rejuvenation, Journal of Cosmetic Dermatology. (2020) 19, no. 11, 2922–2928, 10.1111/jocd.13394. 32243049

[13] Gatti J. E. , Eyelid Phenol Peel: An Important Adjunct to Blepharoplasty, Annals of Plastic Surgery. (January 2008) 60, no. 1, 14–18, 10.1097/sap.0b013e31805003aa, 2-s2.0-39849098325. 18281788

[14] Soon S. L. , Wambier C. G. , Rullan P. R. et al., Phenol-Croton Oil Chemical Peeling Induces Durable Improvement of Constitutional Periorbital Dark Circles, Dermatologic Surgery. (2023) 49, no. 4, 368–373, 10.1097/dss.0000000000003708. 36735802

[15] Starkman S. J. and Mangat D. S. , Chemical Peel (Deep, Medium, Light), Facial Plastic Surgery Clinics of North America. (2020) 28, no. 1, 45–57, 10.1016/j.fsc.2019.09.004. 31779941

[16] Soleymani T. , Lanoue J. , and Rahman Z. , A Practical Approach to Chemical Peels: A Review of Fundamentals and Step-by-Step Algorithmic Protocol for Treatment, The Journal of Clinical and Esthetic Dermatology. (2018) 11, no. 8, 21–28. PMC6122508 30214663

